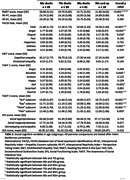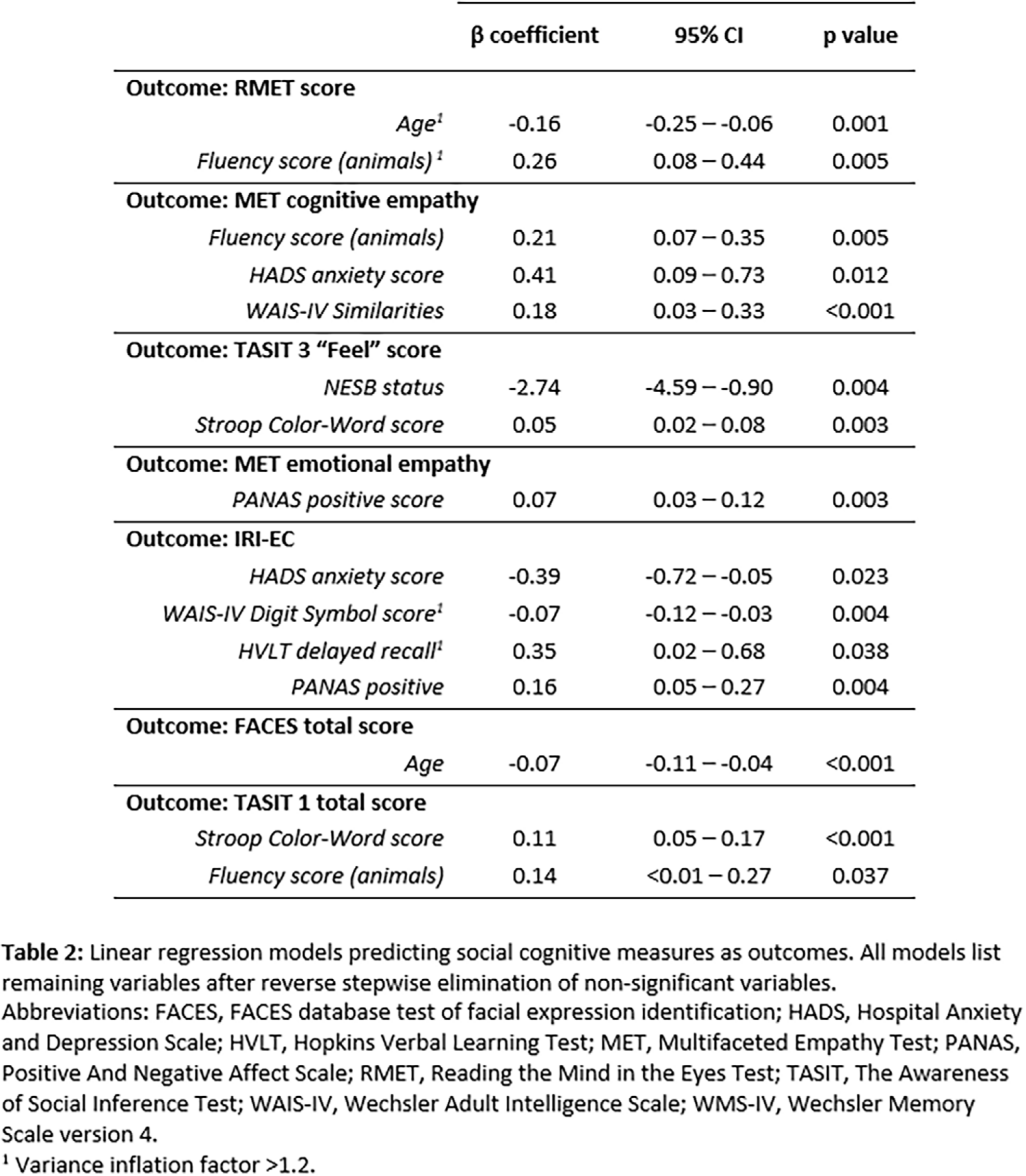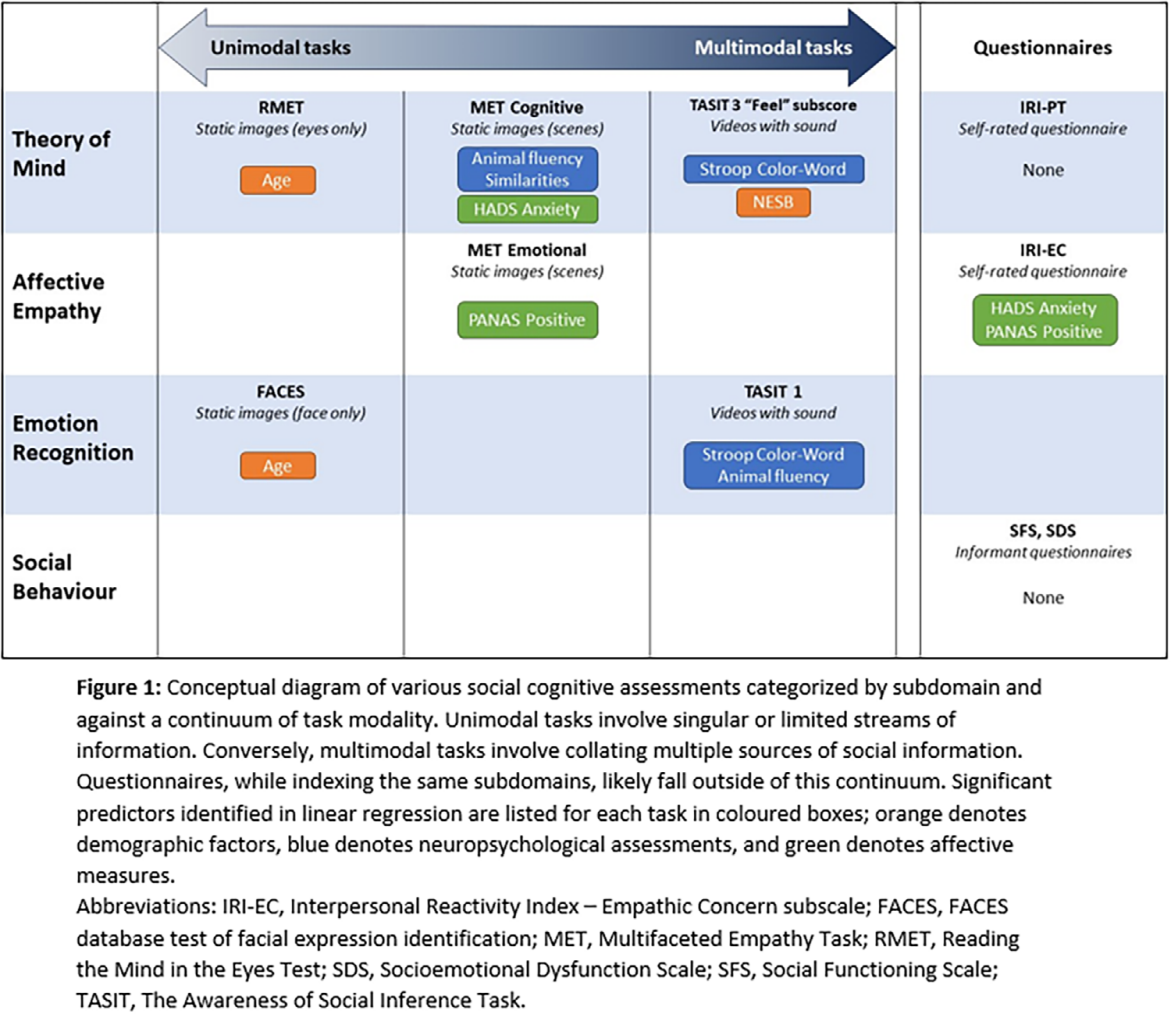# Influence of ageing, cognitive factors, and task modality on social cognitive performance in nondemented older adults

**DOI:** 10.1002/alz.092023

**Published:** 2025-01-03

**Authors:** Russell J Chander, Sarah A Grainger, Karen A Mather, Rhiagh Cleary, Nicole A. Kochan, Julie D Henry, Perminder S. Sachdev

**Affiliations:** ^1^ Centre for Healthy Brain Ageing (CHeBA), University of New South Wales, UNSW Sydney, NSW Australia; ^2^ University of Queensland, Brisbane, QLD Australia; ^3^ Neuroscience Research Australia, Sydney Australia; ^4^ Neuropsychiatric Institute, Prince of Wales Hospital, Randwick, NSW Australia

## Abstract

**Background:**

Social cognition is crucial to optimal social functioning outcomes in older adults, with implications for overall health and wellbeing. Moreover, poor social cognition is a diagnostic criterion for neurocognitive disorders (NCDs). Prior work has studied the social cognitive subdomains (theory of mind (ToM), affective empathy, emotion recognition, and social behaviour) and found mild cognitive impairment and dementia to be associated with poorer performance in specific tasks and informant‐reported changes respectively. These patterns in NCDs need to be distinguished from normal age‐related changes, and more information is needed to ascertain what factors predict social cognitive decline in healthy ageing.

**Method:**

132 non‐demented participants [mean MMSE 29.23 (SD 0.99)] aged 60 to 100 underwent comprehensive social cognitive assessments that varied based on modality of information (Figure 1), alongside neurocognitive assessments and affective questionnaires. Participants were divided based on decades to study age‐related changes. Linear regression models identified significant predictors of social cognition performance amongst demographic, neuropsychological assessment scores, affective scores, and social networks.

**Result:**

Age was associated with poorer performance in ToM tasks, specifically the Reading the Mind in the Eyes Test (RMET), Multifaceted Empathy Test (MET) Cognitive subscale, and The Awareness of Social Inference Test (TASIT) part 3, and emotion recognition tasks, specifically the FACES task and TASIT 1, for expressions of anger, disgust, and sadness (Table 1). In regression models (Table 2), age was a significant predictor of RMET and FACES performance. In MET cognitive and both TASITs, neuropsychological tasks, mainly executive function, were predictive. Poorer executive function predicted performance for identifying unspoken emotions (“Feel” subscore) in TASIT 3. MET Emotional and the self‐rated Interpersonal Reactivity Index (IRI) Empathic Concern subscale was associated with less anxiety and more positive emotions at assessment.

**Conclusion:**

In ToM and emotion recognition, unimodal task performance was associated with age and some cognitive factors, and multimodal task performance was largely associated with executive function. Performance in affective empathy tasks was linked to affective measures. Poor performance in multimodal tasks likely reflects executive dysfunction in consolidating multiple streams of social information, while some declining performance in unimodal tasks may be normative to ageing.